# Supplementary material for: Enhanced sensitivity via non-Hermitian topology
Source: Light Sci Appl. 2025 Jan 1;14:6. doi: 10.1038/s41377-024-01667-z (PMC11688499; doi:10.1038/s41377-024-01667-z)
Supplement: Supplementary file 1 — Supplementary Material [file 41377_2024_1667_MOESM1_ESM.pdf]

Supplementary Information for Enhanced sensitivity via non-Hermitian topology

Midya Parto[1,2,3†], Christian Leefmans[4,†], James Williams[1], Robert M. Gray[1], Alireza Marandi[1,4,*]

[1]Department of Electrical Engineering, California Institute of Technology, Pasadena, CA 91125, USA.

[2]Physics and Informatics Laboratories, NTT Research, Inc., Sunnyvale, California 94085, USA.

[3]CREOL, The College of Optics and Photonics, University of Central Florida, Orlando, FL, USA.

[4]Department of Applied Physics, California Institute of Technology, Pasadena, CA 91125, USA.

[†]These authors contributed equally

[*]marandi@caltech.edu

1 Sensitivity analysis of the NTOS

In this section, we explain the principle of operation of NTOS and its exponentially enhanced sensitivity.

We consider the Hatano-Nelson lattice shown in Fig. S1. The eigenstates of this lattice can be found using the non-Hermitian Hamiltonian formalism

$$-\frac{d}{dt}|\psi\rangle = H_{\mathrm{HN}}|\psi\rangle, \tag{1}$$

where H_{HN} is defined in the Eq. 1 of the main text and is equivalent to the following N by N matrix operator

$$H_{\mathrm{HN}} = \begin{bmatrix} 0 & t_L & 0 & \cdots & 0 \\ t_R & 0 & t_L & \cdots & 0 \\ \vdots & \vdots & \vdots & \vdots & \vdots \\ \Gamma t_L & 0 & 0 & \cdots & 0 \end{bmatrix}. \tag{2}$$

Note that in Eq. 1 the eigenvalues represent decay rates since the couplings implemented in our experiments are dissipative couplings [1]. Under open boundary conditions, i.e. $\Gamma = 0$, this Hamiltonian supports the zero right and left eigenstates with zero eigenvalue $E_0 = 0$ given by

$$|\psi_0\rangle_R = \begin{bmatrix} 1 \\ 0 \\ \alpha \\ 0 \\ \alpha^2 \\ 0 \\ \vdots \\ \alpha^k \end{bmatrix}, \langle\psi_0|_L = \begin{bmatrix} 1 & 0 & 1/\alpha & 0 & 1/\alpha^2 & \cdots 1/\alpha^k \end{bmatrix}, \tag{3}$$

where $\alpha = -t_R/t_L$ and we are assuming that the number of lattice elements is odd $N = 2k+1$. On the other hand, under perturbed boundary conditions, i.e. when $\Gamma \neq 0$, using biorthogonal first-order perturbation theory one can find the shift in the eigenvalue associated with the zero

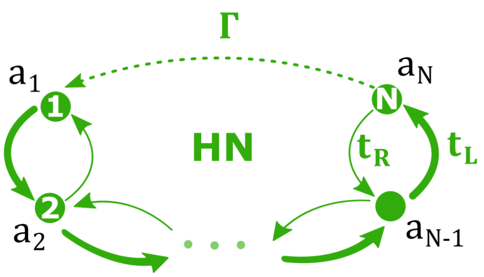

Figure 1: **Analytical solution for the perturbed zero state in the Hatano-Nelson lattice.**

eigenstate as

$$\Delta E_n = \frac{\langle\psi_0|_L \Delta H|\psi_0\rangle_R}{\langle\psi_0|_L\psi_0\rangle_R} \propto \Gamma(\sqrt{1/\alpha})^N. \tag{4}$$

This latter equation clearly indicates the exponential enhancement of the sensitivity defined as $S \equiv \partial\Delta E/\partial\Gamma$ with respect to the number of lattice elements N in a Hatano-Nelson lattice, in accordance with experimental results presented in Fig. 5 of the main text.

As mentioned in the main text, a remarkable property of the NTOS is the fact that its response is robust with respect to unwanted perturbations in the HN lattice, i.e. disorder in the bulk couplings within the lattice. To show this, we estimate the shift in the eigenvalue of the zero eigenstate in response to off-diagonal perturbation in the the Hamiltonian of Eq. 1 of the main text.

$$\Delta E \approx \frac{1}{N}\begin{bmatrix}1 & 0 & 1/\alpha & 0 & 1/\alpha^2 & \cdots 1/\alpha^k\end{bmatrix}\begin{bmatrix}0 & \Delta t_{12} & 0 & \cdots & 0\\ \Delta t_{21} & 0 & \Delta t_{23} & \cdots & 0\\ \vdots & \vdots & \vdots & \vdots & \vdots\\ 0 & 0 & 0 & \cdots & 0\end{bmatrix}\begin{bmatrix}1\\0\\\alpha\\0\\\alpha^2\\0\\\vdots\\\alpha^k\end{bmatrix} = 0. \tag{5}$$

Hence, the shift in the eigenvalue of the zero eigenstate tends to remain insensitive with respect to the unwanted fluctuations of the couplings within the Hatano-Nelson model which do not affect the boundary conditions.

Let us now consider the sensitivity of NTOS to diagonal elements in the Hamiltonian matrix which represent fluctuations in the losses associated with different pulses in the cavity. To examine this, we estimate the shift in the eigenvalue of the zero eigenstate in response to diagonal perturbations in the the Hamiltonian of Eq. 1 of the main text using first-order perturbation theory.

$$\Delta E \approx \frac{1}{N} \begin{bmatrix} 1 & 0 & 1/\alpha & 0 & 1/\alpha^2 & \cdots 1/\alpha^k \end{bmatrix} \begin{bmatrix} 0 & \cdots & 0 & \cdots & 0 \\ \vdots & \vdots & \vdots & \vdots & \vdots \\ 0 & \cdots & \delta\gamma_m & \cdots & 0 \\ \vdots & \vdots & \vdots & \vdots & \vdots \\ 0 & \cdots & 0 & \cdots & 0 \end{bmatrix} \begin{bmatrix} 1 \\ 0 \\ \alpha \\ 0 \\ \alpha^2 \\ 0 \\ \vdots \\ \alpha^k \end{bmatrix} = \frac{[1-(-1)^m]}{N}\delta\gamma_m. \tag{6}$$

In other words, the shift in the eigenvalue of the zero mode in response to a small shift in the diagonal elements of the Hamiltonian is zero if the lattice site number is even. If the lattice site number associated with the diagonal perturbation is odd (worst case), then the shift in the zero eigenvalue will be an attenuated version of the perturbation. Hence, we see that although the response of NTOS is not completely zero to the loss fluctuations associated with the pulses, it tends to be suppressed and not amplified by NTOS. To further confirm this response, we numerically simulated the changes in the eigenvalue of the zero mode of the Hamiltonian H_{HN} in Eq. 1 in response to a change in the diagonal element associated with the first and last lattice sites with a magnitude of $\delta\gamma$. Figure S2 shows the simulation results, which clearly indicate that the NTOS sensitivity to such perturbations is small and decreases as the size of the lattice

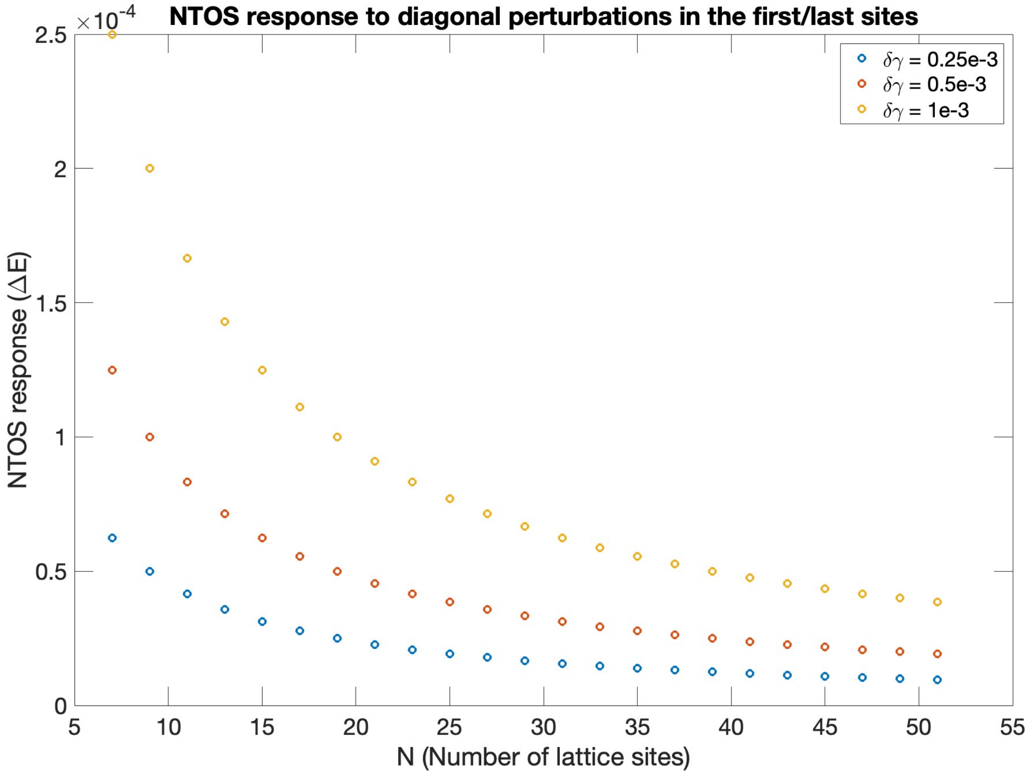

Figure 2: **NTOS response to diagonal perturbations in the first/last sites.**

N grows.

Finally, we consider the response of the NTOS to the input (Γ) in the presence of diagonal disorder. In particular, we numerically evaluate the dynamics described by the perturbed Hamiltonian $H_{\text{HN}} + \Delta H$ when a diagonal disorder of $\delta\gamma$ is also applied to the first/last lattice site. Figure S3 summarizes these results. As shown here, it is clear that the exponentially enhanced response of NTOS persisits even in the presence of on-site (diagonal) disorders, as long as these disorders are smaller than the total response at the output of the NTOS, i.e. $\delta\gamma < \Delta E$.

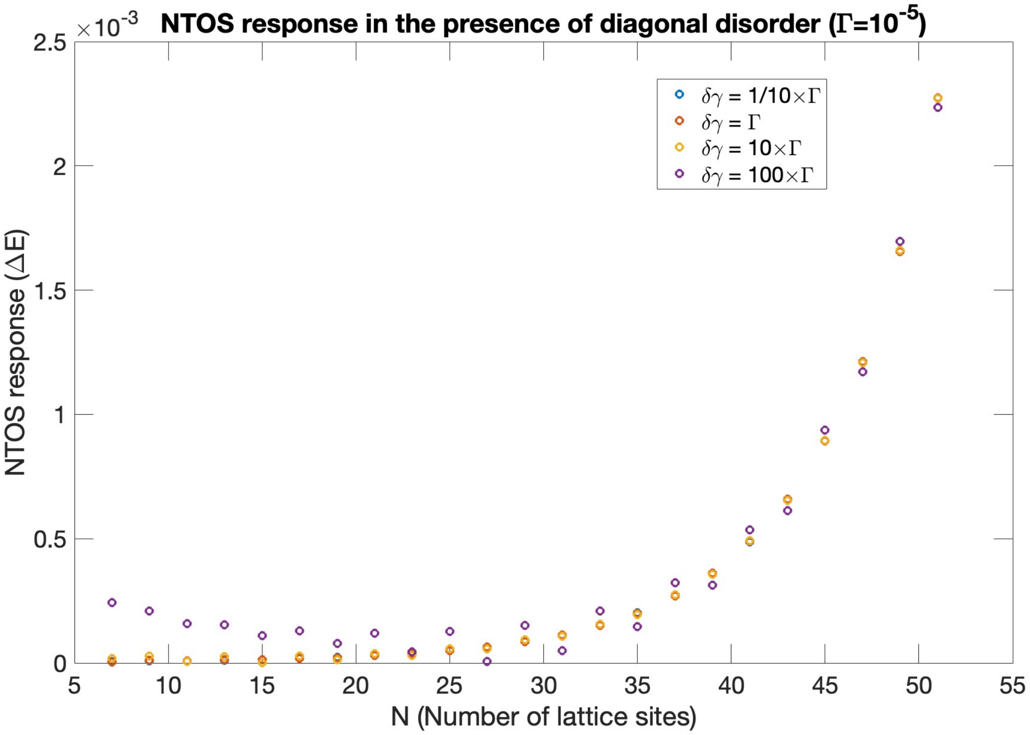

Figure 3: **NTOS response in the presence of on-site disorders.**

2 Experimental Setup

We demonstrate non-Hermitian topological sensors (NTOS) using the time-multiplexed photonic resonator network shown in Fig. S4. As has been discussed previously, such networks are excellent architectures for studying lattice models in temporal synthetic dimensions [1], as they can enable the realization of multidimensional synthetic lattices, long-range couplings, and tunable boundary conditions. In this work, we leverage the ability to implement long-range couplings and tunable boundary conditions to realize NTOS in our time-multiplexed network.

At a high level, the time-multiplexed network in Fig. S4 consists of a main cavity and three optical delay lines. The main cavity can support up to 74 optical pulses separated by a repetition period $T_{\mathrm{R}} \approx 4\ \mathrm{ns}$. Meanwhile, the lengths of the three delay lines are designed so that the $\pm 1 T_{\mathrm{R}}$ delay lines implement nearest-neighbor Hatano-Nelson (HN) couplings between the pulses, and the $+(N-1)T_{\mathrm{R}}$ delay line couples the "first" pulse to the "last" pulse in an N site HN lattice. In our experiments, the coupling produced by the $+(N-1)T_{\mathrm{R}}$ delay line acts as a perturbation on an N-site HN lattice with open boundary conditions (OBCs). Adjusting the length of the $+(N-1)T_{\mathrm{R}}$ delay line enables us to study how perturbations effect HN lattices of different sizes.

We inject pulses into our time-multiplexed network using the mode-locked laser (MLL) shown in Fig. S4. This MLL generates femtosecond pulses at a repetition period of $\sim 4\ \mathrm{ns}$, and we stretch the pulses to widths of $\sim 5\ \mathrm{ps}$ using a Channel 34 dense wavelength-division multiplexing (DWDM) filter. After stretching the pulses, we send them through a 90:10 splitter. Light from the 10% port goes directly to a $600\ \mathrm{MHz}$ photodetector, and the output of this detector passes through a $300\ \mathrm{MHz}$ low-pass filter to generate a $\sim 250\ \mathrm{MHz}$ sinusoid. This sinusoid acts as a clock for the FPGA that drives the modulators in our time-multiplexed network. We find that using a clock signal derived directly from our optical pulses improves the synchroniza-

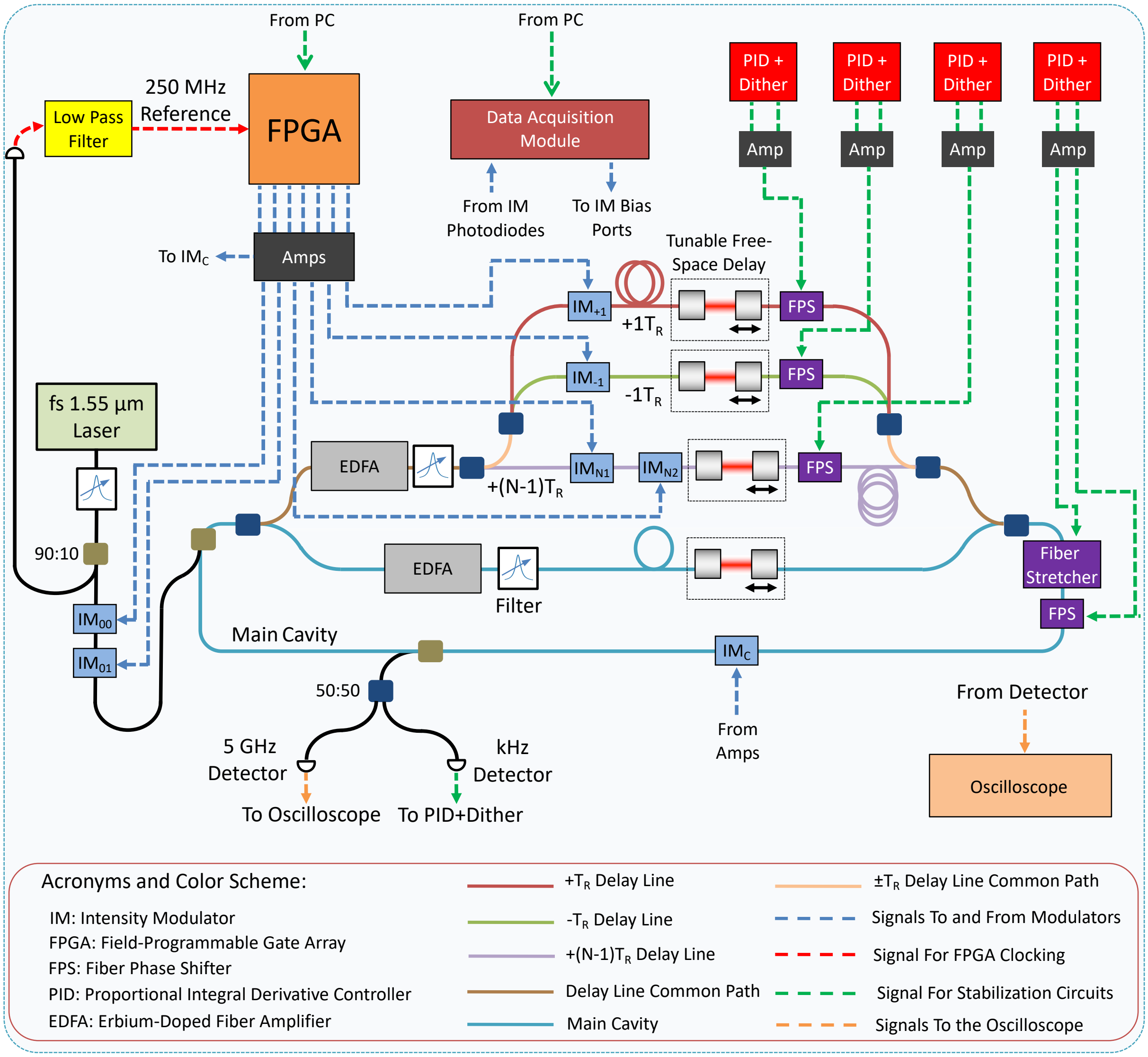

Figure 4: **Schematic of the experimental setup used to realize NTOS.**

tion between the electronics and photonics in our experiment. Meanwhile, light from the 90% port passes through two intensity modulators (IMs), labeled IM_{00} and IM_{01}. These modulators prepare the pulse patterns that we inject into the network, which in this work, are the right zero-modes of HN lattices of various sizes. We use IM_{00} to carve the desired zero-mode from the incoming pulse train, while we use IM_{01} to improve the extinction ratio for sites in the pulse pattern that are supposed to have zero amplitude.

After passing through IM_{00} and IM_{01}, the pulses pass through another 90:10 splitter, and 10% of the light is injected into the network. Within the network, a 50:50 splitter divides the pulses between a common delay line path and the continuation of the main cavity. We add erbium-doped fiber amplifiers (EDFAs) to both of these paths. In the main cavity, the EDFA allows us to partially compensate for the roundtrip losses. In the delay line path, the EDFA enables us to increase the coupling strengths between the pulses. After each EDFA, we add Channel 34 DWDM filters to remove amplified spontaneous emission (ASE) noise.

After the filter, the light in the common delay line path is divided at another 50:50 splitter. Half of the light goes into the $+(N-1)T_{\mathrm{R}}$ delay line, and the other half goes to yet another 50:50 splitter, where it is divided between the $\pm 1T_{\mathrm{R}}$ delay lines. In each delay line, there is a tunable free space delay, which enables us to adjust the lengths of the delay lines; at least one IM, which allows us to control coupling strengths during an experiment; and a fiber phase shifter (FPS), which we use to stabilize the delay lines relative to the main cavity. After passing through these elements, we recombine the delay lines with more 50:50 splitters before finally recombining all of the delay lines with the main cavity at a final 50:50 splitter.

After recombining the delay lines and main cavity, the light passes through a fiber stretcher, a FPS, and an IM before finally reaching the second 90:10 splitter in the main cavity. The fiber stretcher and the FPS are used to stabilize the main cavity, while the IM (IM_{C}) is used to "Q-switch" the cavity during our experiment. In particular, we find that it is easier to stabilize

our cavity and delay lines if the finesse of the main cavity is lower, so we use IM_{C} to reduce the cavity finesse when we lock our system prior to an experiment. During the experiment, we increase the cavity finesse to lower the decay rate of the cavity. The second 90:10 splitter in the main cavity outputs 10% of the light in the main cavity. This output is divided with a 50:50 splitter between a slow (kHz) detector, which is used for stabilization, and a 5 GHz detector, which is used to capture the relative pulse amplitudes on our oscilloscope. The 90% of the light that remains in the main cavity returns to the input 90:10 splitter.

In addition to the FPGA, which drives the IMs in our experiment, there are several other electronic components to our setup. First, we use a dedicated data acquisition module to set the bias voltages of our modulators. This module sets the voltages of IM_{00}, $IM_{\pm 1}$, and IM_C. Due to the limited number of channels on our data acquisition module, we use a separate power supply (not shown in Fig. S4) to set the biases for IM_{N1}, IM_{N1}, and IM_{01}. Second, we use Red Pitaya STEMLabs to perform lock for the delay lines and the main cavity in phase. These off-the-shelf modules contain built in Pound-Drever-Hall (PDH) locking capabilities [2], we use them in conjunction with custom-built PCBs, the FPSs in the delay lines, and the FPS and fiber stretcher in the main cavity to stabilize our system.

3 Calibration

Here, we briefly describe the calibration procedure used to prepare the input waveforms and the pulse-to-pulse couplings used in our experiment.

To construct the input waveforms using IM_{00} and IM_{01}, we view the output of these two IMs directly on an oscilloscope. Leaving IM_{01} biased to maximum throughput, we apply a voltage ramp to IM_{00} to generate a curve of the output optical power as a function of the applied voltage. We use this calibration curve to generate a first-pass input waveform for IM_{00}. Recall that this waveform is supposed to generate the right HN zero-mode from the input pulse

train. For our next step in the calibration, we iteratively improve our first-pass waveform. We drive IM_{00} with the current waveform and average the optical response over several traces. In software, we compare this average trace to the expected HN zero-mode, and we update our driving waveform accordingly. We continue this procedure until we attain the desired accuracy of the input waveform. Note that, during this step in the calibration, we also re-bias IM_{01} to minimum throughput and apply the waveform that this modulator will see during the experiment. The waveform on IM_{01} opens the modulator to maximum throughput when a site in the HN zero-mode is supposed to be nonzero, and it leaves the modulator at minimum throughput when a site in the HN zero-mode is supposed to have zero amplitude.

To calibrate the IMs in the $\pm 1T_{\mathrm{R}}$ delay lines, we first bias the IMs to minimum throughput. We then iteratively tune the waveform amplitudes applied the two modulators until the ratio of the coupling strength produced by the $-1T_{\mathrm{R}}$ delay line to that produced by the $+T_{\mathrm{R}}$ delay line is $\sqrt{2}$. Note that we only drive these modulators when they need to produce couplings between the pulses. In particular, while our cavity can support up to 74 pulses, the HN lattices studied in our experiments are much smaller. Therefore, we only drive the IMs when they produce nearest-neighbor couplings in the HN lattice under study. For other (empty) time slots, we leave the modulators at minimum throughput so that we reduce spurious couplings between the lattice and the surrounding time slots.

After calibrating the IMs in the $\pm 1T_{\mathrm{R}}$ delay lines, we calibrate the $+(N-1)T_{\mathrm{R}}$ delay line to introduce a perturbation between the first and final sites of the HN lattice under study. Because the perturbation is small, it is not possible to calibrate the $+(N-1)T_{\mathrm{R}}$ delay line by viewing the throughput of the delay line on our oscilloscope. Therefore, we resort to measuring the average power through the $+(N-1)T_{\mathrm{R}}$ delay line relative to the average power through the $+1T_{\mathrm{R}}$ delay line. To do this, we connect a power meter to one of the unused ports of the 50:50 splitter after the $\pm 1T_{\mathrm{R}}$ delay lines recombines with the $+(N-1)T_{\mathrm{R}}$ delay line. We then configure IM_{+1} so

that the $+1T_{\mathrm{R}}$ delay line outputs a constant stream of pulses whose amplitudes correspond to the coupling strength that will be used during the experiment. Measuring the average power of this pulse train from the $+1T_{\mathrm{R}}$ delay line tells us by how much we must attenuate the power in the $+(N-1)T_{\mathrm{R}}$ in order to introduce the desired perturbation.

With all of the other delay lines blocked, we next observe the average output power of the $+(N-1)T_{\mathrm{R}}$ delay line on the power meter. We begin to attenuate this delay line by biasing both IM_{N1} and IM_{N2} to maximum throughput then detuning the coupling in the free-space delay shown in Fig. S4. In this manner, we attenuate the delay line to a point where we can achieve the desired perturbation by properly reducing the throughput of the IMs but where we can also still lock the $+(N-1)T_{\mathrm{R}}$ delay line when the IMs are set to maximum throughput. At this point, we measure the power out of the delay line once more and calculate how much further we need to attenuate the power to achieve the desired perturbation.

We distribute the remaining attenuation between the IM_{N1} and IM_{N2}. With one of the modulators biased to maximum throughput, we tune the bias of the other modulator to introduce a certain level of attenuation. We then record the bias voltage at which this attenuation is achieved. We repeat this procedure for both modulators to achieve the full degree of attenuation. Although the bias of these modulators is not actively stabilized, we verify that the throughput of the IMs is stable enough that the degree of attenuation does not vary substantially over the time scale of our experiments. After determining the proper bias voltage for each modulator, we set the bias voltages for both modulators. Note that, because we do not actively modulate the modulators in this delay line, the coupling in the $+(N-1)T_{\mathrm{R}}$ delay line is always on. In experiment, we find that this fact does not have a substantial effect on the observed results.

4 Experimental Procedure and Data Analysis

4.1 Experimental Procedure

After calibrating the IMs in our network, we are ready to begin our experiment. Prior to running an experiment, we prepare our network in the so-called "locking cycle," in which we lock the main cavity and all of the delay lines in phase. Recall that the modulators in the $\pm 1T_{\mathrm{R}}$ delay lines are biased to minimum, while the modulators in the $+(N-1)T_{\mathrm{R}}$ delay line are biased to introduce the desired perturbation between the first and final pulses of the HN lattice. Therefore, to lock the delay lines, we drive the IMs to enable sufficient throughput for our locking electronics to function properly. Furthermore, we drive the intracavity IM IM_{C} (which is biased to maximum throughput) to reduce the finesse of the cavity. As was mentioned earlier, we find that reducing the finesse of our cavity during the locking cycle facilitates locking the main cavity and the delay lines simultaneously.

After locking the network, we initiate a program that triggers our FPGA and runs our experiment. Upon triggering the FPGA, the output of the FPGA switches the network from the locking cycle to the experiment cycle. At the beginning of the experiment cycle, we use IM_{C} to suppress any residual light that might be in the cavity from the locking cycle. Then we stop driving IM_{C} to maximize the finesse of the cavity. After we stop driving IM_{C}, we inject the right zero-mode of the HN lattice under study into the network for 10 roundtrips, which enables the zero-mode to build up to a steady state. On the $10th$ roundtrip, we initiate the couplings in the $\pm 1T_{\mathrm{R}}$ delay lines. We then stop injecting light into the cavity, and we observe the decay of the HN zero mode in the presence of the perturbation introduced by the $+(N-1)T_{\mathrm{R}}$ delay line.

Ideally, in the absence of the perturbation, the HN zero-mode would decay at the same rate as an uncoupled pulse in our network. Therefore, we can measure the effect of the perturbation by measuring the decay rate of the HN zero-mode relative to the decay rate of an uncoupled

pulse. We do this in our experiment by injecting an additional pulse into one of the unused time slots of our cavity at the same time that we inject the HN zero-mode. Our delay lines are programmed so that this pulse is uncoupled from the other time slots in the cavity, and, therefore, this pulse provides a reference for the decay rate of the measured zero mode.

Additionally, we inject a second reference pulse into another unused time slot of the main cavity, and we allow this reference pulse to couple to its direct nearest-neighbors. This reference pulse allows us to observe the reliability of our nearest-neighbor couplings and helps to ensure that the timings of the delay lines and the injected state are properly synchronized.

4.2 Data Analysis

As illustrated in Fig. 3 of the main text, the state of the time-multiplexed network in every roundtrip is represented by the amplitudes of the pulses within the cavity time slots which define different site numbers in the Hatano-Nelson lattice. In our experiments, we record these time traces for 100 instances and use the average of these traces to measure the state of the network $|\psi(mT_{\text{RT}})\rangle$ in successive roundtrips $m = 1, 2, ...$, where T_{RT} represents the roundtrip time of the optical cavity. We then project this state into the left eigenstate of the unperturbed HN model to define $P(m) = \langle\psi_0|_L\psi(mT_{\text{RT}})\rangle$. From here, we estimated the decay rate of $P(m)$ per cavity roundtrip to measure the response of NTOS defined as the quantity $|\Delta E \times T_{\text{RT}}|$ that is reported in Fig. 4 of the main text.

5 Analytical solution for the perturbed zero state

In this section, we present analytical results to evaluate the exact eigenvalue of the zero eigenstate associated with a finite Hatano-Nelson lattice with perturbed boundary conditions. This situation is schematically shown in Fig. S1.

Using coupled mode theory, the evolution of the field amplitudes within different lattice sites in

this lattice is governed by:

$$\begin{aligned}\frac{da_n}{dt} &= t_R a_{n-1} + t_L a_{n+1}, 2 < n < N-1 \\ \frac{da_1}{dt} &= t_L a_2, \\ \frac{da_N}{dt} &= t_R a_{N-1} + \Gamma a_1.\end{aligned} \tag{7}$$

Using the ansatz $a_i(t) = A_i e^{Et}$,the solution to these coupled differential equations can be given by

$$A_n = A_1 \sum_{m=0}^{m=\lceil n/2\rceil-1} \binom{n-m-1}{n-2m-1} (-1)^m E^{n-(2m+1)} \frac{t_R^m}{t_L^{n-m-1}}, 1 < n. \tag{8}$$

Assuming $N = 2k+1$ and by substituting Eq. 8 into the last equation in Eq. 7 one finds

$$f(E) \equiv \sum_{m=0}^{m=k} \binom{2k+1-m}{2k+1-2m} (-1)^m E^{2k+1-2m} \frac{t_R^m}{t_L^{2k+1-m}} - \Gamma = 0. \tag{9}$$

Assuming the perturbed eigenvalue is real, Eq. 9 must have a real root such that $f(E(\Gamma)) = 0$ where $-t_L < E(\Gamma) < t_L$. Therefore, in accordance to Bolzano's theorem we expect that

$$f(t_L) f(-t_L) < 0. \tag{10}$$

Meanwhile, $f(t_L) = (-x)^k B_k(-1/x) - \Gamma$, where $x = t_R/t_L$ and B_k denote the Morgan-Voyce polynomials which are related to the Chebyshev polynomials of the second kind via $B_k(x) = U_k(1 + x/2)$ [3]. The conditions defined by the Eq. 11 then translate into

$$\Gamma < x^k |U_k(1 - 1/x)| = \Gamma_C, \tag{11}$$

which clearly indicate that the conditions for the perturbed finite HN lattice to have a real root limits the boundary coupling Γ to smaller values that scale exponentially with the size of the finite lattice.

6 Sensitivity analysis of trivial and Hermitian topological lattices

In this section we theoretically analyze the sensitivity of trivial and Hermitian topological lattices with respect to their associated boundary conditions. Let us first consider a trivial lattice shown in Fig. S5a. Here, the evolution of the field amplitudes within different lattice sites in this lattice is governed by:

$$\begin{aligned} & i\frac{da_n}{dt} + ta_{n-1} + ta_{n+1} = 0, 2 < n < N-1 \\ & i\frac{da_1}{dt} + \Gamma a_N + ta_2 = 0, \\ & \frac{da_N}{dt} + \Gamma a_1 + ta_{N-1} = 0. \end{aligned} \tag{12}$$

When $\Gamma = 0$, using the ansatz $a_m = (A'e^{imq} + B'e^{-imq})e^{-iEt}$ one finds the eigenvalues to be $E = -2tcos(q)$ where $q = k\pi/(N+1), k = 1, 2, ..., N$ defines discrete quasi-momenta in the lattice. From here, the field amplitudes can be obtained as $a_m = Asin(mq)e^{-iEt}$. Based on these results, we can now estimate the change in the eigenvalues as a result of the change in the boundary conditions $\Gamma \neq 0$:

$$|\Delta E_n| = |\frac{\langle\psi_n|\Delta H|\psi_n\rangle}{\langle\psi_n|\psi_n\rangle}| = |(-1)^{k+1} \times 4\Gamma/N \sin^2 q| < 4\Gamma/N. \tag{13}$$

This is consistent with the results presented in the Fig. 5 of the manuscript.

Next, we consider a one-dimensional Su-Schrieffer-Heeger (SSH) model as an example of a Hermitian topological lattice (Fig. S5b). Similar analysis in this case shows that the shift in the eigenvalue of the defect state can be estimated again using first-order perturbation theory

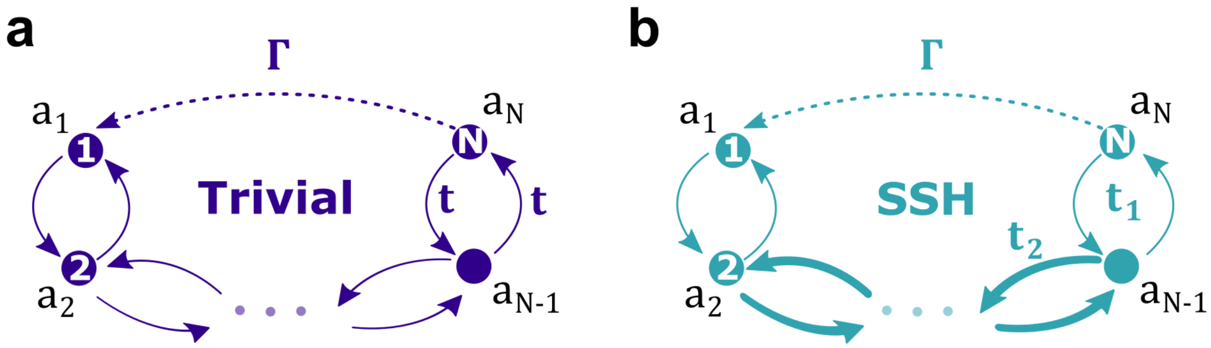

Figure 5: **Sensitivity analysis of trivial and Hermitian topological lattices. a**, Trivial lattice and **b**, the SSH model as an example of a Hermitian topological lattice.

$$\Delta E_0 = \frac{\langle\psi_0|\Delta H|\psi_0\rangle}{\langle\psi_0|\psi_0\rangle} \approx \frac{1-e^{-2\alpha}}{1-e^{-2N\alpha}} \times \begin{bmatrix}1 & 0 & -e^{-\alpha} & 0 & e^{-2\alpha} & 0\cdots 0\end{bmatrix} \begin{bmatrix}0 & \cdots & \Gamma \\ \vdots & \vdots & \vdots \\ \Gamma & \cdots & 0\end{bmatrix} \begin{bmatrix}1 \\ 0 \\ -e^{-\alpha} \\ 0 \\ e^{-2\alpha} \\ 0 \\ \vdots \\ 0\end{bmatrix} = 0. \tag{14}$$

Here, $e^{-\alpha} = t_1/t_2$. This insensitivity is consistent with the results presented in Fig. 5 of the main text associated with a Hermitian topological lattice.

7 Sensor response and noise

In this section, we first provide theoretical analysis in line with [4, 5] regarding the response of our implementation of NTOS to an external probe that confirms its exponential sensitivity. Here, we assume that the dynamics of the time-multiplexed resonators is governed by the perturbed Hamiltonian $H = H_{\text{HN}} + \Delta H$:

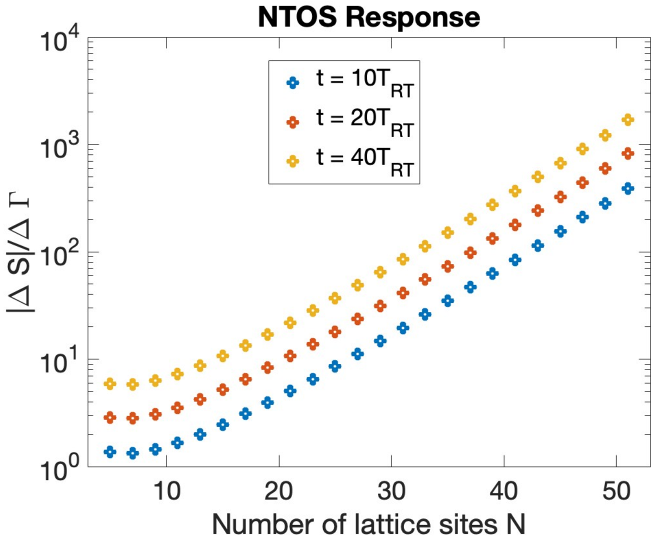

Figure 6: **Theoretical analysis of the NTOS response probed by an appropriate input state.**

$$\frac{d}{dt}|\psi(t)\rangle = H|\psi(t)\rangle, \tag{15}$$

assuming the initial probe injected into the system is equal to $|\psi(t)\rangle = |\psi_0\rangle_R$, i.e. the right eigenstate of the unperturbed Hamiltonian. In order to generate a readout signal $S(t)$, the state of the system is projected into the left eigenstate of the unperturbed Hamiltonian, i.e. $S(t) = \langle\psi_0|_L\psi(t)\rangle$. Finally, we consider the logarithm of the change in the signal as the response of our sensor $\Delta S = log(S(\Gamma, t)) - log(S(\Gamma = 0, t))$. Figure S6 summarizes these results, thus confirming the exponential sensitivity enhancement of the NTOS implemented in our study.

In order to experimentally characterize the noise in our setup, we run the experiment by injecting the zero eigenstate of the unperturbed Hamiltonian H_{HN} with $N = 23$ into the cavity and block the $(N-1)T$ delay line to set the perturbation $\Gamma = 0$. We then experimentally measure the fluctuations in the intensity of the last pulse #23 that represents the last lattice site in our resonator array (please see Fig. S7). In our experiments, we measure these fluctuations by

calculating the standard deviations in the intensities for an ensemble of measurements, resulting in a value of $\delta I_{23} \approx 7 \times 10^{-3} [a.u.]$. Assuming a conventional linear sensing model, the minimum detectable perturbation in the coupling Γ from pulse $\#1$ to pulse $\#23$ would be $2\Gamma_{min} \times T_{\mathrm{RT}} > 2\delta I_{23}/I_1$. Hence, using a conventional sensing method the minimum detection limit set by the thermal noise in our experiments is estimated as $\Gamma_{min} \times T_{\mathrm{RT}} \approx 6 \times 10^{-3}$. As mentioned in the main text, our proposed NTOS effectively amplifies small perturbations in the coupling Γ, hence providing an enhanced response. Our experimental results presented in Fig. 4 of the main text shows that using NTOS with $N = 23$ pulses, we were able to measure perturbations as small as $\Gamma \times T_{\mathrm{RT}} \approx 1.5 \times 10^{-3}$. Such improvements in the detection limit is also consistent with the sensitivities reported in Fig. 5 of the manuscript.

References

1. Leefmans, C. *et al.* Topological dissipation in a time-multiplexed photonic resonator network. *Nature Physics* **18,** 442–449 (2022).
2. Black, E. D. An introduction to Pound–Drever–Hall laser frequency stabilization. *Am. J. Phys.* **69,** 79–87 (2001).
3. Merikoski, J. K. Regular polygons, Morgan-Voyce polynomials, and Chebyshev polynomials. *Notes on Number Theory and Discrete Mathematics* **27,** 79–87 (2021).
4. Langbein, W. No exceptional precision of exceptional-point sensors. *Physical Review A* **98,** 023805 (2018).
5. Budich, J. C. & Bergholtz, E. J. Non-Hermitian Topological Sensors. *Physical Review Letters* **125,** 180403 (2020).

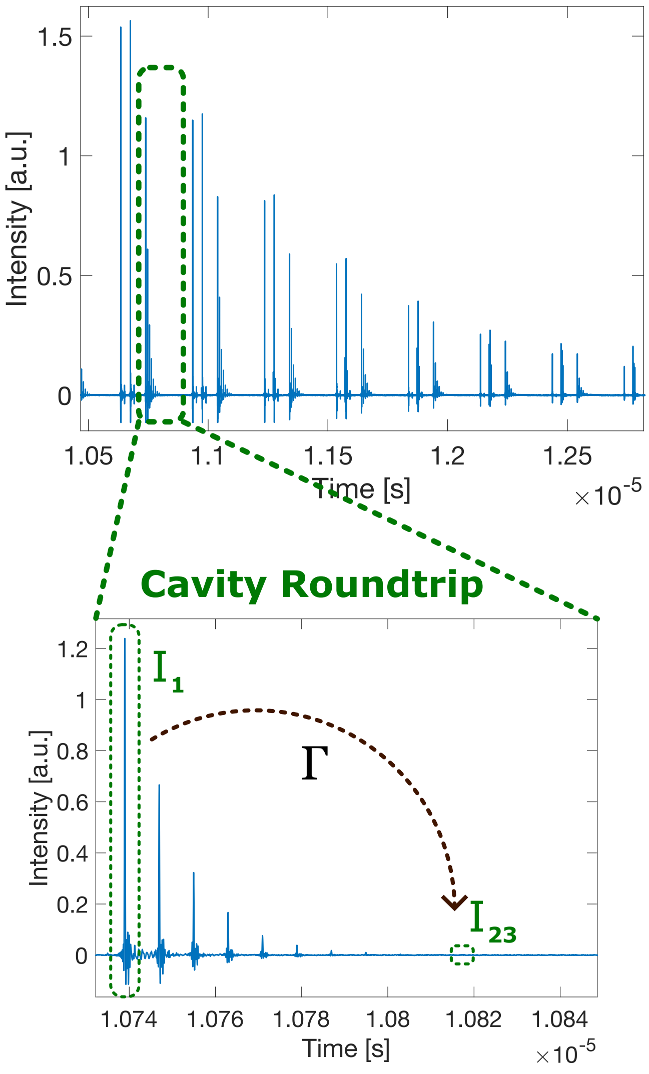

Figure 7: **Estimating the end-to-end coupling Γ in a cavity.**
